# Effects of Scoparone on differentiation, adhesion, migration, autophagy and mineralization through the osteogenic signalling pathways

**DOI:** 10.1111/jcmm.17476

**Published:** 2022-07-07

**Authors:** Kyung‐Ran Park, Bomi Kim, Joon Yeop Lee, Ho‐Jin Moon, Il Keun Kwon, Hyung‐Mun Yun

**Affiliations:** ^1^ Gwangju Center Korea Basic Science Institute (KBSI) Gwangju Korea; ^2^ National Development Institute of Korean Medicine Gyeongsan Korea; ^3^ Department of Dental Materials, School of Dentistry Kyung Hee University Seoul Korea; ^4^ Medical Device Research Center, Medical Science Research Institute Kyung Hee University Medical Center Seoul Korea; ^5^ Department of Oral and Maxillofacial Pathology, School of Dentistry Kyung Hee University Seoul Korea

**Keywords:** *Artemisia capillaris*, autophagy, differentiation, mineralization, osteoblast, SCOP

## Abstract

Scoparone (SCOP), an active and efficient coumarin compound derived from *Artemisia capillaris* Thunb, has been used as a traditional Chinese herbal medicine. Herein, we investigated the effects of SCOP on the osteogenic processes using MC3T3‐E1 pre‐osteoblasts in in vitro cell systems. SCOP (C_11_H_10_O_4_, > 99.17%) was purified and identified from *A. capillaries*. SCOP (0.1 to 100 μM concentrations) did not have cytotoxic effects in pre‐osteoblasts; however, it promoted alkaline phosphatase (ALP) staining and activity, and mineralized nodule formation under early and late osteogenic induction. SCOP elevated osteogenic signals through the bone morphogenetic protein 2 (BMP2)‐Smad1/5/8 pathway, leading to the increased expression of runt‐related transcription factor 2 (RUNX2) with its target protein, matrix metallopeptidase 13 (MMP13). SCOP also induced the non‐canonical BMP2‐MAPKs pathway, but not the Wnt3a‐β‐catenin pathway. Moreover, SCOP promoted autophagy, migration and adhesion under the osteogenic induction. Overall, the findings of this study demonstrated that SCOP has osteogenic effects associated with cell differentiation, adhesion, migration, autophagy and mineralization.

## 1 INTRODUCTION

The *Artemisia capillaris* Thunb is a traditional herbal medicine which possesses remarkable therapeutic and protective effects in hepatic diseases.^1^, ^2^, ^3^ *A. capillaris* contains coumarin and flavonoid compounds, which possess anti‐oxidant, anti‐inflammatory, anti‐cancer and anti‐osteoporosis activities.^4^, ^5^, ^6^, ^7^, ^8^ Scoparone (6,7‐dimethoxycoumarin) (SCOP), which is primarily found in Artemisia plant roots, is a coumarin compound and has pharmacological properties relating to vasorelaxation or immunosuppression.^9^, ^10^ Recently, it has been discovered that SCOP suppresses inflammatory responses by inhibiting PI3K‐Akt signalling in chondrocytes, suggesting that SCOP may be a therapeutic compound in degenerative and bone diseases such as osteoarthritis.^11^

Mesenchymal stem cells (MSCs) and pre‐osteoblasts differentiate, adhere and migrate to specific regions, leading to the bone‐specific protein secretion and matrix mineralization.^12^, ^13^, ^14^ Osteoblast differentiation and maturation help multicellular organisms maintain bone tissue homeostasis and control skeletal development, formation and remodelling.^13^, ^15^ Osteoblast malfunction induces bone formation abnormalities, resulting in low bone mass and fragile fractures in bone diseases.^16^ By regulating bone metabolism, anabolic drugs for bone diseases can improve osteoblast differentiation and bone formation; however, there are issues relating to drug safety and cost.^16^, ^17^, ^18^, ^19^, ^20^ Currently, osteogenic compounds are of increasing interest, as these compounds have relatively fewer side effects and may be used for a longer period of time than previously used drugs for bone diseases.^21^, ^22^ Thus, it is worth investigating the identification of osteogenic compounds and their pharmacological effects on osteoblast differentiation, migration, adhesion and mineralization.

The purpose of this study was to examine the osteogenic roles of SCOP of dry, aboveground *A. capillaris* tissue in differentiation, migration, adhesion, autophagy and mineralization in in vitro cell systems using MC3T3‐E1 pre‐osteoblasts that have been studied in osteoblast differentiation. Our findings demonstrated that SCOP has bone‐forming activities, suggesting potential roles as an osteogenic compound.

## 2 METHODS

### 2.1 Plant material

*Artemisia capillaris* Thunb was purchased from Korean medicine Omniherb (www.omnishop.co.kr). The P149 voucher specimen was deposited in the Natural Products Bank (NIKOM). Column chromatography (CC) was conducted using silica gel (Merck, Darmstadt, Germany). ^13^C Nuclear magnetic resonance (NMR) and ^1^H NMR spectra were analysed using JEOL ECX‐500 spectrometer (JEOL Ltd.). Agilent 1260 series (Agilent Technologies) with a C18 column (Phenomenex, United States of America Synergi 10 μ Hydro‐RP 80A, 10 μm, 4.6 mm × 250 mm) was used for high‐performance liquid chromatography (HPLC).

### 2.2 Culture and differentiation

MC3T3‐E1 pre‐osteoblasts (#CRL‐2593) were obtained from the ATCC (Manassas, VA). Pre‐osteoblasts were grown at 37°C, 5% CO_2_ and 95% air under a humidified atmosphere in α‐MEM without L‐AA (WELGEME, Inc.) containing 10% foetal bovine serum (FBS) and 1× Gibco antibiotic‐antimycotic (Thermo Fisher Scientific). OS containing 10% FBS, 1× Gibco antibiotic‐antimycotic, 50 μg/ml L‐AA (Sigma‐Aldrich) and 10 mM β‐GP (Sigma‐Aldrich) with SCOP was used to stimulate osteoblast differentiation. 0.1, 1, 5, 10, 20, 30, 40, 50 and 100 mM SCOP stocks were prepared with 100% dimethyl sulfoxide (DMSO) and diluted to a final concentration (1:1000 dilution, 0.1% DMSO); 0.1% DMSO was treated as a control. During osteoblast differentiation, OS was changed every 2 days as described previously.^15^

### 2.3 Cell viability

Cell viability was analysed using MTT solution (Sigma‐Aldrich). Absorbance was detected at 540 nm using the Multiskan GO Microplate Spectrophotometer (Thermo Fisher Scientific) as described previously.^23^

### 2.4 ALP staining and activity assays

For ALP staining assay, the ALP reaction solution was treated according to the manufacturer's protocol (Takara Bio Inc., Japan), as described previously.^24^ For ALP activity assay, cell lysates were obtained using alkaline phosphatase activity colorimetric assay kit (Biovision). The activity was detected at 405 nm using the spectrophotometer (Thermo Fisher Scientific) as described previously.^24^

### 2.5 ARS staining assay

Cells were stained with 2% ARS solution (pH 4.2) (Sigma‐Aldrich) for 10 min. Stains were captured using a scanner, and absorbance was detected at 590 nm using the spectrophotometer (Thermo Fisher Scientific) as described previously.^24^

### 2.6 Western blot analysis

Osteogenic‐ and autophagic‐protein levels were detected using Western blot analysis as described previously.^24^, ^25^ The antibodies used were: RUNX2 (1:2000, #12556), p‐Smad1/5/8 (1:1000, #13820), p‐p38 (1:2000, #9211S), p38 (1:2000, #9212), p‐ERK1/2 (1:3000, #9101), ERK1/2 (1:3000, #9102), p‐JNK (1:2000, #9251), JNK (1:2000, #9252), GSK3β (1:1000, #12456), p‐GSK3β (1:1000, #9336), β‐catenin (1:1000), Wnt3a (1:1000, #2721), LC3A/B (1:1000, #12741), Beclin1 (1:1000, #3495) from Cell Signaling Technology (Beverly, MA, USA); BMP2 (1: 500, #CSB‐PAO9419AORb) from CUSABIO (Houston); β‐actin (1:2000, #sc‐47,778) from Santa Cruz Biotechnology (Santa Cruz); HRP‐secondary antibodies (1:20,000) from Jackson ImmunoResearch (West Grove).

### 2.7 Immunofluorescence assay

Nuclear RUNX2 expression was analysed using immunofluorescence as described previously.^24^ The following antibodies were used: RUNX2 (1:400, #12556, Cell Signaling Technology), and secondary antibody (Alexa‐Fluor 488, 1:500, Invitrogen). DAPI (4′,6‐diamidino‐2‐phenylindole) solution was used (Sigma‐Aldrich) for nucleus stains.

### 2.8 DAPGreen autophagy detection assay

Autophagy Detection Kit (Dojindo) was used for detecting autophagosome formation as described previously.^25^

### 2.9 Cell adhesion assay

Adhesion was analysed using Matrigel‐coated plates (Corning Life Sciences). The crystal violet‐stained cells were monitored under a light microscope, and absorbance was detected at 540 nm using the spectrophotometer (Thermo Fisher Scientific) as described previously.^15^

### 2.10 Cell migration assay

Migration across ECM was carried out using Boyden chamber in Matrigel (Corning Life Sciences)‐coated nuclear pore filters. The images obtained under a light microscope were quantified as previously described.^26^

### 2.11 Statistical analysis

Prism Version 5 program from GraphPad Software, Inc. was used for statistical analysis. Significance in *p* < 0.05 was analysed by using a one‐way anova with post hoc analysis, and the differences were assessed by the Bonferroni test. All values were reported as mean ± S.E.M.

## 3 RESULTS

### 3.1 Purification of SCOP from the extracts of *A. capillaris* dry aboveground part

**FIGURE 1:**
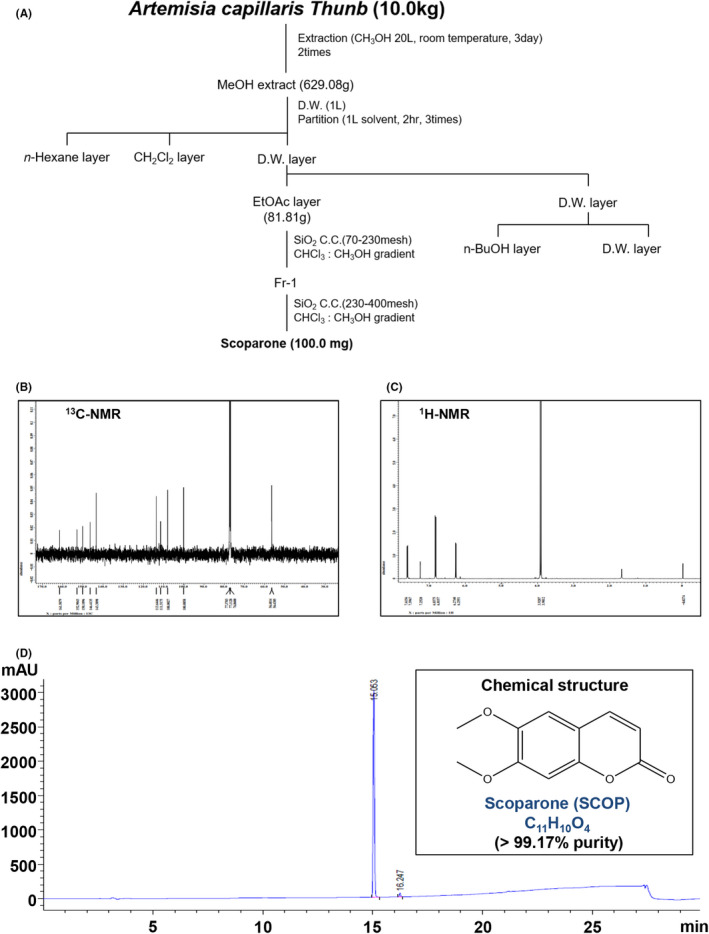
Isolation of scoparone (SCOP) from *Artemisia capillaris*. (A) Schematic of the method used to isolate and purify SCOP from *A. capillaris*. (B and C) ^13^C‐NMR (125 MHz, CDCl_3_) (B) and ^1^H‐NMR (500 MHz, CDCl_3_) (C) spectra of SCOP. (D) HPLC of purified SCOP (D), and its chemical structure, purity, and molecular formula ((D) inset)

The dry aboveground part of *A. capillaris* Thunb (10 kg) was extracted in MeOH at room temperature for 3 days. The crude extract (629.08 g) was suspended in DW and organic solvents. EtOAc soluble fraction (81.81 g) was subjected to silica gel CC, and fraction 1 was subjected to silica gel CC eluted with mixtures of CHCl3‐MeOH to afford compound 1 (SCOP, 100.0 mg) (Figure 1A). 125 MHz, CDCl3, ^13^C NMR: δ 160.5 (C‐2), 152.5 (C‐7), 149.4 (C‐6), 145.8 (C‐9), 144.3 (C‐4), 112.6 (C‐10), 111.2 (C‐3), 108.9 (C‐5), 100.0 (C‐8), 56.2 (OMe), 55.9 (OMe) (Figure 1B). 500 MHz, CDCl3, ^1^H‐NMR: δ 7.94 (1H, d, J = 9.5 Hz, H‐4), 7.24 (1H, s, H‐5), 7.06 (1H, s, H‐8), 6.29 (1H, d, J = 9.5 Hz, H‐3), 3.86 (3H, s, ‐OCH3), 3.80 (3H, s, ‐OCH3) (Figure 1C). Figure 1D displays SCOP's HPLC and structure (>99.17% purity; molecular formula: C_11_H_10_O_4_) (Figure 1D).

### 3.2 SCOP accelerates early and late osteoblast differentiation

**FIGURE 2:**
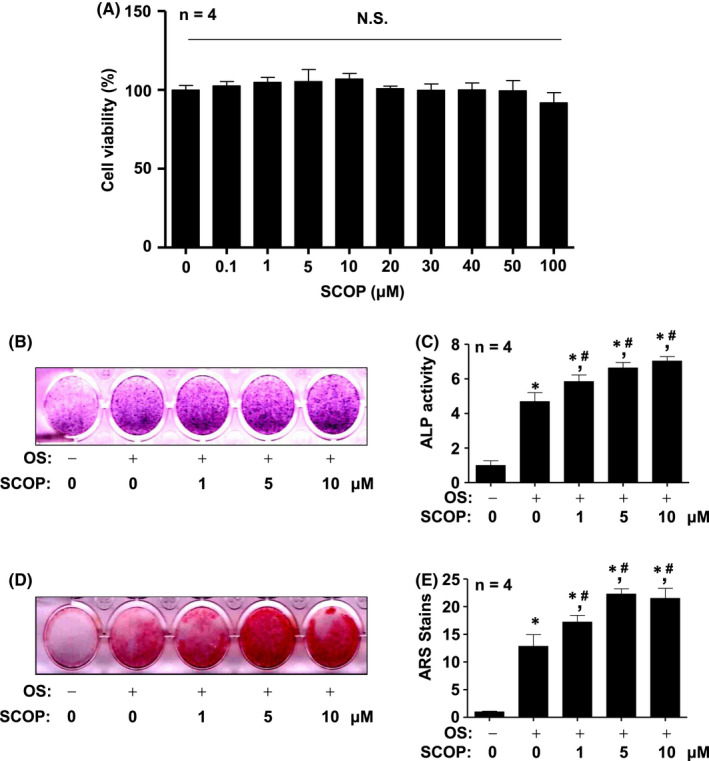
Cytotoxic and osteogenic effects of SCOP on pre‐osteoblasts. (A) Viability was detected using the MTT assay after treatment with SCOP (0.1 ~ 100 μM) for 24 h in pre‐osteoblasts. (B and C) The early differentiation was detected at 7 days using the ALP staining (B) and activity (C) assays after incubation in OS with SCOP (1–10 μM). (D and E) The late differentiation was detected to analyse the mineralization using the ARS staining assay after incubation in OS with SCOP (1–10 μM) for 21 days (D), and the stains were quantitatively analysed using a spectrophotometer (E). Data are represented as mean ± S.E.M. *p < 0.05 and ^#^ *p* < 0.05 indicate statistical significance compared with the control and OS, respectively

Cell viability (%) was measured using the MTT assay to investigate whether SCOP induces cytotoxicity after treating SCOP for 24 h. As shown in Figure 2A, SCOP showed no cytotoxic effects against pre‐osteoblasts at 0.1–100 μM (Figure 2A). Next, we induced osteoblast differentiation using OS with SCOP (1–10 μM) for 24 h to examine whether SCOP possesses osteogenic effects. Alkaline phosphatase (ALP) staining was performed to detect pre‐osteoblast early differentiation. ALP staining images were captured by a scanner, which revealed that SCOP elevated osteoblast differentiation at 7 days (Figure 2B). ALP enzymatic activity was detected by a spectrophotometer, which validated SCOP‐mediated osteoblast differentiation (Figure 2C). An Alizarin Red S (ARS) staining assay was used to explore whether SCOP modulates matrix mineralization by the late osteoblast differentiation. We used a scanner to detect the matrix mineralization at 21 days after inducing osteoblast differentiation using OS with SCOP (110 μM). The matrix mineralization was generated, which showed that SCOP accelerated late osteoblast differentiation (Figure 2D). The osteogenic effects of SCOP on the differentiation were statistically validated by quantifying ARS staining (Figure 2E). In addition, we found that SCOP increased OPG/RANKL during osteoblast differentiation (Figure S1A).

### 3.3 SCOP promotes canonical BMP2‐Samd1/5/8 signalling and RUNX2 expression

**FIGURE 3:**
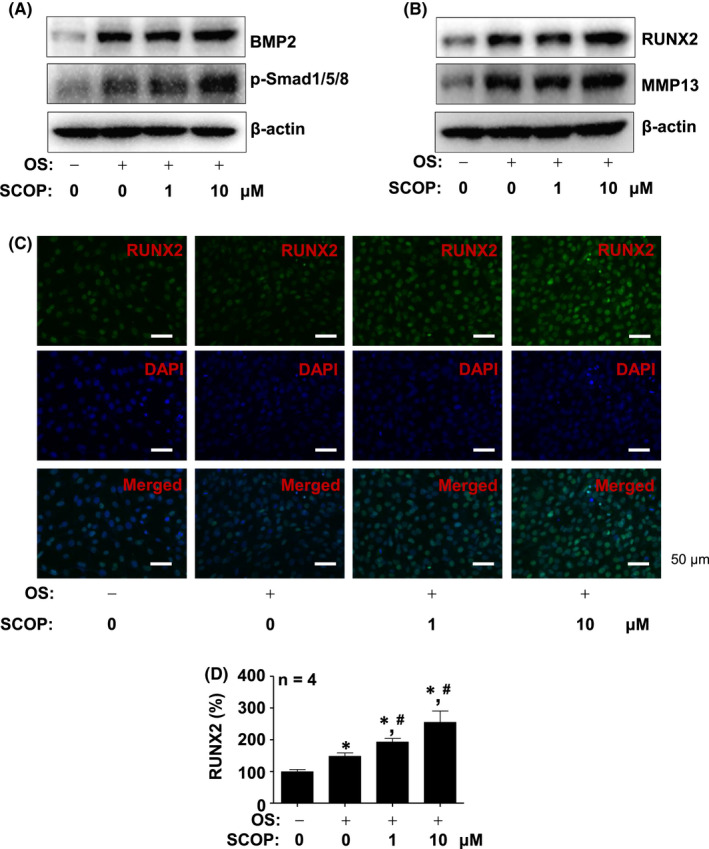
Effects of SCOP on canonical BMP2 signalling and RUNX2. (A) Western Blot analysis of BMP2, phospho‐Smad1/5/8 (p‐Smad1/5/8) and β‐Actin levels. (B) Western Blot analysis of RUNX2, MMP13 and β‐Actin levels. (C and D) Immunofluorescence assay to assess RUNX2 (green) levels in nucleus (blue). Images were detected using a fluorescence microscope. RUNX2 (%) was shown in a bar graph. (D). Data are mean ± S.E.M. *p < 0.05 and ^#^ *p* < 0.05 indicate statistical significance compared with the control and OS, respectively

We further examined the mechanism by which SCOP acts on osteoblast differentiation by detecting canonical BMP2 signalling using Western blot analysis. We observed that SCOP increased intracellular and secreted BMP2 protein levels, and induced Smad1/5/8 phosphorylation, which is a key canonical BMP2 signalling protein (Figure 3A and (Figure S1B). SCOP also promotes RUNX2 protein levels, which is a core transcription factor induced by BMP2 signalling in osteoblast differentiation, followed by MMP13 protein expression, which is a RUNX2‐target protein (Figure 3B). The increased RUNX2 expression in the nucleus in response to SCOP was also validated using an immunofluorescence assay (Figure 3C). In addition, we validated that SCOP‐stimulated early and late osteoblast differentiation was attenuated by a BMP2 inhibitor, Noggin (Figure S1C,D).

### 3.4 SCOP promotes non‐canonical BMP2‐MAPKs signalling and autophagosome formation

**FIGURE 4:**
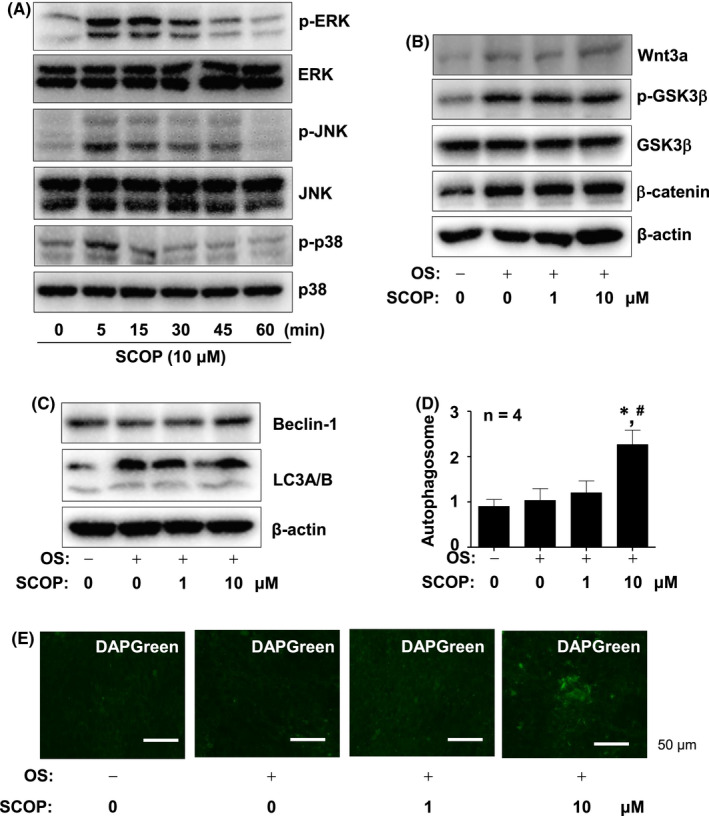
Effect of SCOP on non‐canonical BMP2 signalling, Wnt3a signalling and autophagy. (A) Western Blot analysis of phospho‐p38 (P‐P38), p38, phospho‐JNK (p‐JNK), JNK, phospho‐ERK1/2 (p‐ERK), ERK and β‐Actin levels. (B) Western Blot analysis of Wnt3a, phospho‐GSK3β (p‐GSKβ), GSK3β, β‐catenin and β‐Actin levels. (C) Western Blot analysis of Beclin‐1, LC3A/B and β‐Actin levels. (D and E) Autophagosome formation was analysed and the DAPGreen‐stained autophagosome intensity was shown in a bar graph (D), and images were detected using a fluorescence microscope (E). Data are mean ± S.E.M. *p < 0.05 and ^#^ *p* < 0.05 indicate statistical significance compared with the control and OS, respectively

We subsequently examined whether SCOP affects the BMP2‐mediated non‐canonical pathway. As shown in Figure 4A, SCOP activated the non‐canonical pathway proteins, ERK, p38 and JNK (Figure 4A). We also investigated Wnt3a signalling, which revealed that SCOP did not noticeably affect the signalling proteins, including Wnt3a expression, GSK3β phosphorylation and β‐catenin stabilization (Figure 4B). Furthermore, autophagy signalling was investigated, and Western blot analysis revealed that Beclin1 and LC3A/B were increased by 10 μM SCOP, but not by 1 μM SCOP (Figure 4C). In addition, autophagy was observed by using a DAPGreen autophagosome formation assay, which revealed that 10 μM SCOP increased autophagy through the increased formation of autophagic vacuoles (Figure 4D,E).

### 3.5 SCOP promotes osteoblast adhesion and migration

**FIGURE 5:**
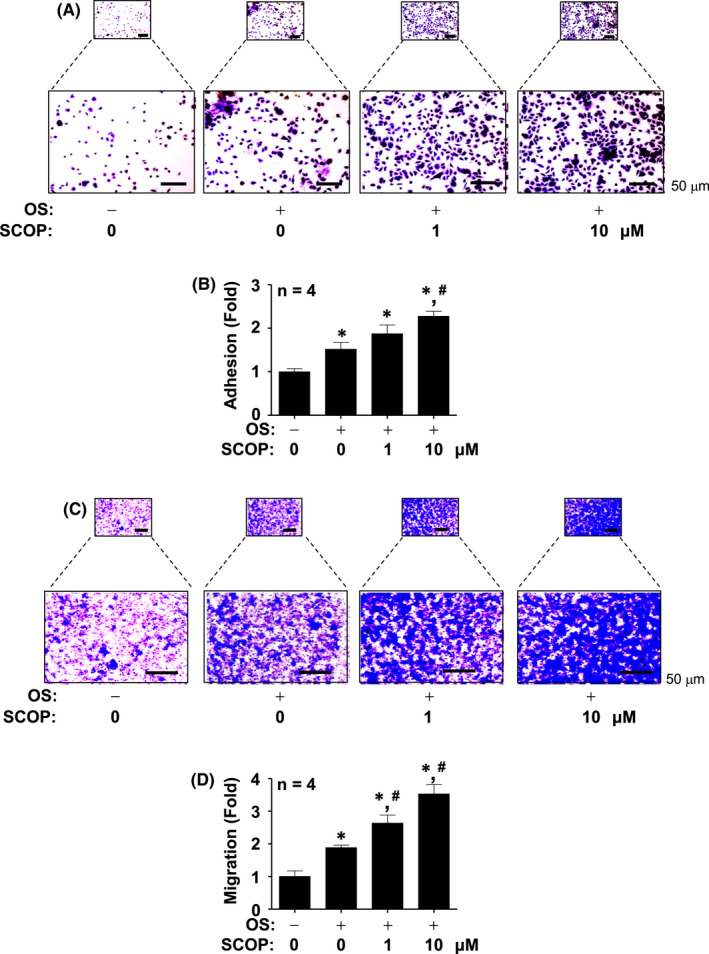
Effect of SCOP on osteoblast adhesion and migration (A and B) Adhesion was analysed on ECM‐coated plates, and images were detected using a light microscope. (A) Adhesion was shown in a bar graph (B). (C and D) Migration was analysed using Boyden chamber in ECM‐coated membrane, and images were detected using a light microscope (C). Migration was shown in a bar graph (D). Data are mean ± S.E.M. *p < 0.05 and ^#^ *p* < 0.05 indicate statistical significance compared with the control and OS, respectively

We finally explored whether SCOP influences osteoblast migration and adhesion in osteogenic processes. Adhesion experiments performed using Matrigel‐coated culture plates showed that SCOP marginally accelerated cell adhesion at 1 μM, and by a significant amount at 10 μM (Figure 5A,B). A Boyden chamber assay also showed that SCOP significantly increased osteoblast migration across the membrane coated by Matrigel (Figure 5C,D).

## 4 DISCUSSION

Osteoblasts control bone tissue homeostasis throughout life, and the damage and loss of osteoblasts can lead to skeletal diseases.^21^, ^27^ Herein, we demonstrated the activities of SCOP on MC3T3E‐1 pre‐osteoblasts, which are commonly utilized for researching in vitro osteogenic processes.^28^ Osteoblast differentiation increases ALP, while osteoblast maturation results in bone matrix mineralization, which are well‐known early and late osteoblast differentiation markers, respectively.^27^, ^29^, ^30^ Herein, we found that SCOP enhances ALP and bone matrix mineralization during osteogenic processes. We also demonstrated that SCOP accelerates osteoblast adhesion and migration. Osteoblast differentiation, migration and adhesion to the blood, bone marrow, periosteum induce bone‐specific protein secretion and bone matrix mineralization to produce bone tissue.^12^, ^13^, ^14^ Our findings, based on the previous papers and present results, indicate that SCOP has osteogenic effects by promoting differentiation, adhesion, migration and mineralization.

Osteogenic processes are tightly regulated through complex networks activated by distinct osteogenic signalling molecules. BMP2, a bone morphogenetic factor, stimulates the canonical Smad1/5/8 and the non‐canonical MAPKs pathways, which subsequently control the critical transcription factor RUNX2. These events result in the production of bone‐forming proteins such as *osterix*, collagen and ALP for osteoblast differentiation.^31^, ^32^, ^33^ Herein, SCOP enhances BMP2 and activates Smad1/5/8, ERK, JNK and p38. SCOP also increases RUNX2 expression and increases production of the target protein, MMP13, which is associated with calcification and degradation of extracellular matrix (ECM), as well as cell migration and adhesion during osteoblast differentiation bone repair.^34^, ^35^, ^36^ In osteoblast differentiation, there is a close relationship between the BMP2 and Wnt3a signalling pathways, including the effects of Wnt3a regulating BMP2 expression and its target proteins.^37^, ^38^, ^39^, ^40^, ^41^ However, we found that SCOP did not affect Wnt3a expression, GSK3β phosphorylation or β‐catenin stabilization. Thus, our results suggest that SCOP accelerates differentiation and mineralization by promoting BMP2‐mediated osteogenic signalling.

Autophagy is a self‐degradative mechanism in which cells engulf defective organelles and proteins, allowing metabolic balance to be maintained.^42^ Autophagy‐related components play a role in bone metabolism and bone diseases by regulating the survival and function of bone tissue cells.^43^, ^44^, ^45^, ^46^, ^47^ There is increasing evidence that shows that osteoblast differentiation and maturation are stimulated by autophagic process.^44^, ^48^ It was also reported that endoplasmic reticulum stress is induced by defective autophagy in osteoblasts, resulting in significant bone loss.^49^ Recently, natural compound‐induced autophagy has been shown to enhance osteoblast differentiation and maturation, as well as ameliorate bone diseases.^50^, ^51^, ^52^ Here, we demonstrate that high‐dose SCOP elevates autophagosome formation with increased Beclin‐1 and LC3A/B levels. It was reported that BMP2 induces autophagy to facilitate stem cell differentiation.^53^ MAPK‐induced autophagy prevents MC3T3‐E1 pre‐osteoblasts from entering apoptosis.^54^ Thus, our results suggest that SCOP‐induced BMP2 signalling is also involved in the autophagy pathway in osteogenic processes.

In conclusion, the findings of this study show for the first time that SCOP extracted from *A. capillaris* enhances the osteogenic processes—osteoblast differentiation, adhesion, migration, autophagy and mineralization through the BMP2 signalling pathways. However, in future studies, it will be necessary to compare human and murine osteoblasts, as they may behave differently in different species. Our data suggest that SCOP is a potential coumarin for developing an anabolic medication to improve osteoblast differentiation.

## AUTHOR CONTRIBUTIONS

**Kyung‐Ran Park:** Conceptualization (equal); data curation (equal); formal analysis (equal); investigation (equal); methodology (equal); validation (equal); visualization (equal); writing – original draft (equal). **Bomi Kim:** Investigation (equal); methodology (equal); resources (equal); software (equal); validation (equal). **JoonYeop Lee:** Investigation (equal); methodology (equal); resources (equal); software (equal); validation (equal). **Ho‐Jin Moon:** Resources (equal). **Il Keun Kwon:** Funding acquisition (equal); resources (equal); writing – review and editing (equal). **Hyung‐Mun Yun:** Conceptualization (equal); funding acquisition (equal); project administration (equal); writing – review and editing (equal).

## CONFLICT OF INTEREST

The authors declare that they have no competing interests.

## Supporting information


Figure S1

## Data Availability

The data that support the findings of this study are available from the corresponding author upon reasonable request.
