# Supplementary material for: Effects of Scoparone on differentiation, adhesion, migration, autophagy and mineralization through the osteogenic signalling pathways
Source: J Cell Mol Med. 2022 Jul 7;26(16):4520–9. doi: 10.1111/jcmm.17476 (PMC9357629; doi:10.1111/jcmm.17476)
Supplement: Supplementary file 1 — Figure S1 [file JCMM-26-4520-s001.pdf]

Supplementary Figure 1

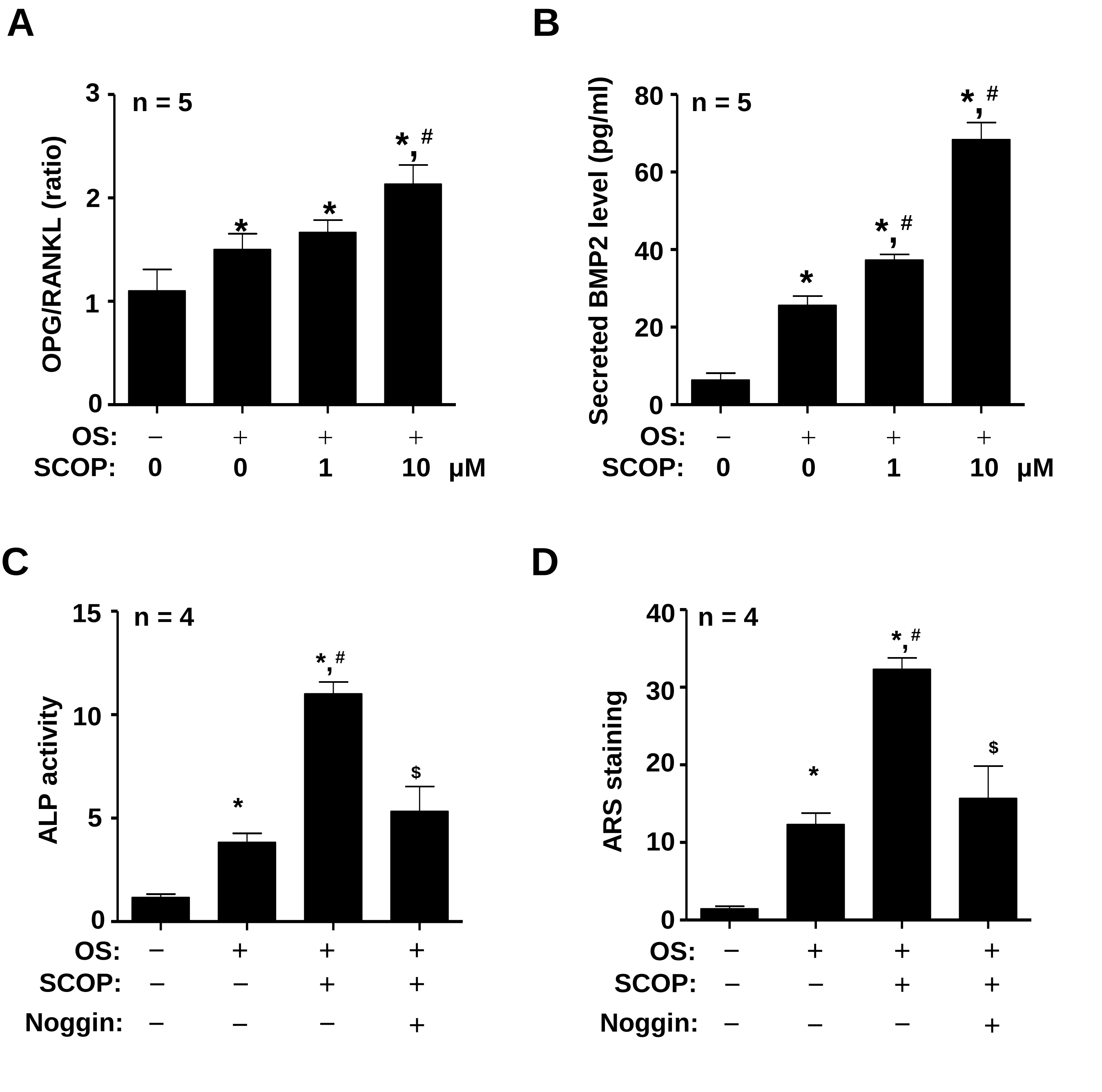

Supplementary Figure 1. Effects of SCOP on OPG/RANKL, Secreted BMP2 level, and osteoblast differentiation by Noggin. **(A)** The cells were differentiated and the RANKL/OPG ratio was measured at 7 days using ELISA. **(B)** Secreted BMP2 level was measured at 7 days using ELISA. **(C, D)** Noggin, a BMP2 inhibitor was treated and ALP at 7 days (C) and mineralization at 21 days (D) were measured and shown in a bar graph. Data are mean ± S.E.M. *, p < 0.05, #, p < 0.05, and $, p < 0.05 indicate statistical significance compared with the control, OS, and OS + SCOP, respectively.
